# Some Principles Relating to Amalgams

**Published:** 1895-09

**Authors:** J. M. Walker


### Monthly Digest.

### SOME PRINCIPLES RELATING TO AMALGAMS.

Reported for the Dental Register by Mrs. J. M. Walker.

In a paper bearing this title, read before the New York Odontological Society,* Dr. E. C. Kirk, noting the marked improvement in the character of amalgam alloys, which, as "everywhere now attainable," "fulfill their function as savers of teeth, under certain conditions, in a manner unapproached by any other material"—finds that this improvement has been largely the outgrowth of the empirical method of study—an investigation addressed principally to the ingredients of the amalgam alloy, and the modifications which these ingredients, in kind, number and amount, exert upon the physical and chemical properties of the resulting amalgam.

Studying the point from a scientific standpoint, Dr. Kirk reaches the conclusion that the amalgam alloys are true chemical compounds, the property of setting or hardening from a plastic mass, the elevation of temperature during this formation, changes in the volume of the mass in setting, all indicating a chemical

* International Dental Journal, April, 1895.

union of some portion of the constituent elements of the amalgam. He concludes that the best results should be attainable in an amalgam the constituents of which are chemically united in atomic ratios; all the affinities in such an amalgam being satisfied, there is no liability of further physical or chemical change. To determine the exact precentage amounts required to effect a combination in atomic ratios, when several metals are used as constituents of an amalgam, is an exceedingly complex problem. The same result is practically attained, however, as pointed out by Dr. Kirk, by utilizing the selective affinity of mercury, supplying sufficient mercury to allow complete solution of the fillings, forming a mass of a soft buttery consistency, which, when rubbed between the fingers shows no trace of solid particles, the elements of the alloy being dissolved in the menstruum. When crystallization has taken place, the excess of mercury (and of uncombined alloy) is gotten rid of by straining through chamois skin by compression with heavy pliers until the mass is left in suitable working condition. With an amalgam thus prepared, the filling should be built to more than full contour and the excess of mercury removed by pressure and absorbing with crystal or sponge gold, pellets of which are rubbed into close contact with the amalgam surface, as long as any whitening of the gold takes place. The filling should then be carefully burnished, and in a very short time given the final polish. Dr. Kirk reviewed the various methods advocated for removing the excess of mercury, and gives the preference to gold, used as described, because of its superior affinity for mercury.

In the discussion, in reply to questions, Dr. Kirk states that he does not leave any of the gold in or on the filling, as this would form a gold amalgam, which is lacking in the qualities for a good filling, and especially is not sufficiently hard for a masticating surface. Dr. Kirk further states in the discussion, that the mercury which is squeezed out through the chamois carries with it portions of the other metals, which being uncombined want to be gotten rid of, so that only that which is in chemical combination shall be utilized.

Dr. Bogue suggested that it is the excess of mercury that causes the discoloration of amalgam fillings, and cited the case of two fillings of the same mix put in the same mouth at the same sitting. If one has the mercury removed by pressure and absorption with gold, and the other be allowed to set without this treatment, the first filling will be hard and firm and white and accurately adapted to the walls, while the other will discolor and be granular, with a tendency to curl up at the edges.

Dr. Kirk thinks the result is due, not to the mercury *per se*, but to the mercury *plus* what it holds dissolved in it. The same fillings mixed with the expressed mercury will not give a combination at all like the original mixture.

Dr. Bogue considers amalgam a material, which, put in with matrices, malleted to press the mercury to the surface and then having the mercury taken off with gold, are good things to contour with, having a surface whiter "than a discolored tooth," which will set before the patient leaves the chair, and which will stay white if the materials are right.

Dr. S. G. Perry uses amalgam as described and has "never found anything else that will compare with it." The crystal gold does not adhere, and cannot easily be made to do so, but it does take away the excess of mercury and he is "willing to pay the extra cost of the gold in that form to be able to take away the excess of mercury in such a quick and satisfactory mannner."

It leaves the filling so hard that if there is such a thing as the "spheroidal tendency" spoken of, it has no opportunity to show itself.

Dr. Henry Burchard agrees with Dr. Kirk that the solution of metals in mercury and subsequent crystallization is a chemical process, and that unless the solvent be present in sufficient amount to insure perfect solution the resulting filling is "an indefinite mass in which we cannot accurately determine the effects of action and interaction."

Dr. Geo. S. Allan wished to thank Dr. Kirk for bringing out so strongly the fact that it is right and according to chemical laws, to prepare the amalgam so that there may be an excess of mercury, the squeezing out of which carries with it a certain amount of alloy that would be injurious. The mercury unites with definite amounts of alloy to form definite compounds any excess whether mercury or alloy remains only as an adulteration.

AMALGAM—ITS USE FROM A PRACTICAL STANDPOINT.

Dr. W. E. Halsey, in a paper bearing the above title, read before the Second District Dental Society, New York,* considers the practical value of a knowledge of the metals entering into the composition of the dental alloys, and their relative proportions in giving an understanding of why one amalgam is superior to another in the most desirable attributes ; how to secure the special feature most needed ; how to rectify defects, etc.

With such knowledge, properly applied, there would be less abuse and misuse of amalgam.

He describes in detail two typical cases illustrating the misuse and the proper use of amalgam, and the combination of zinc-phosphate and amalgam ; also to the combined use in a filling of gutta-percha, zinc-phosphate and amalgam—the first as pulp protector, the second as cavity lining and protection of frail enamel walls, with submarine amalgam to guard against recurrence of decay at the cervical margin, and contour amalgam for edge strength, resistance to attrition from mastication.

By such a combination filling, the pulp is protected from outside influences, weak walls are strengthened and color maintained, recurrence of decay is guarded against by a coppered amalgam at the cervical wall, and lost contour is restored—thus fullfilling the mission of a filling, in tooth salvation.

In the discussion Dr. Van Waert endorses the use of oxyphosphate under amalgam, to secure durability and good color.

Dr. V. H. Jackson mixes amalgam with only enough plasticity to work easily without crumbling, inserts it quickly and burnishes, in from four to ten minutes.

Dr. J. P. Geran thinks that most of the failures in gold filling are due to faulty manipulation and that faulty manipulation is due to the fact that students are not taught to manipulate gold as they were before amalgam came into use. That the introduc-

* Cosmos, April, 1895.

tion of amalgam, cement and gutta-percha, as filling materials, has done much to degrade dentistry.

Amalgam is easier to use than gold, but we should not shirk our duty to our patients because of the draft upon our energies. The consideration should be what is best for the patient not what is easiest for us.

Dr. Halsey closing the discussion said that while he took pride in his gold work, his first consideration was, what material would best preserve the teeth, as they present, and that many teeth can be saved with plastic that cannot be saved with gold.

